# The GPCR A35 regulates fecundity of *Nilaparvata lugens* Stål via juvenile hormone signaling

**DOI:** 10.3389/finsc.2025.1719937

**Published:** 2025-11-26

**Authors:** Minjuan Cao, Di Deng, Ziyue Mao, Zhirou Duan, Yuhang Sun, Xudong Zhao, Linquan Ge

**Affiliations:** 1 College of Plant Protection, Yangzhou University, Yangzhou, China; 2 College of Fine Arts and Design, Yangzhou University, Yangzhou, China; 3 Yantai Forestry Bureau, Yantai Forest Resources Monitoring and Protection Service Center, Yantai, China

**Keywords:** G protein-coupled receptors, *Nilaparvata lugens*, fecundity, juvenile hormone, signal transduction

## Abstract

The brown planthopper, *Nilaparvata lugens* is a major rice pest in Asia, with its high fecundity contributing to recurrent outbreaks. G protein-coupled receptors (GPCRs) are a critical class of transmembrane proteins in insects that sense diverse extracellular and intracellular signals and regulate a wide range of physiological processes. In this study, we characterized GPCR A35 and its function in *N. lugens* fecundity. Expression profiling revealed that GPCR A35 was highly enriched in female heads and fat bodies, with peak levels in females at 4 days post-eclosion. RNAi-mediated the silencing of *GPCR A35* in fifth-instar nymphs by 57–60%, and was effectively delivered to female adults, resulting in a 14.8% reduction in juvenile hormone (JH) titer and marked downregulation of JH biosynthetic and signaling genes, including *HMGCR* (−60.1%), *FPPS* (−57.0%), *JHAMT* (−52.7%), *Met* (−24.2%), and *Kr-h1* (−78.3%). Silencing of *GPCR A35* further decreased *Vg* and *VgR* expression by 82.1% and 72.9% in females at 4 days post-eclosion, reduced protein contents in fat body and ovaries, and impaired ovarian development with fewer mature oocytes. Consequently, female fecundity declined by 51.3%, oviposition duration shortened by 18.5%, and the F1 population growth index decreased by 46.8%. These results demonstrate that GPCR A35 regulates fecundity in *N. lugens* by modulating JH-mediated vitellogenesis and oogenesis, providing a novel molecular target for RNAi-based green pest control.

## 1 Introduction

G protein-coupled receptors (GPCRs) are a critical class of transmembrane proteins in insects that sense diverse extracellular and intracellular signals through their seven-transmembrane helix architecture and transduce these signals into intracellular responses, thereby regulating a wide range of physiological processes (1, 2). As central components of the insect neuroendocrine system and signaling networks, GPCRs are widely distributed across the central nervous system, digestive system, reproductive organs, and sensory structures, where they play essential roles (3). Primarily, GPCRs mediate neurotransmitter and hormonal signaling by recognizing and responding to various neuropeptides, and hormones, and further regulating development, molting, reproduction, and metabolic homeostasis (1, 3–5). Generally, GPCRs are categorized using two widely adopted classification frameworks: the A–F system and the GRAFS system (3). The A–F system categorizes receptors into six groups (A–F) based on sequence homology and functional similarity (6). Among these, Class A, or the “rhodopsin-like” family, is the most abundant; Class B represents the “secretin receptor” family; Class C includes metabotropic glutamate receptors; Class D corresponds to fungal pheromone receptors; Class E encompasses cAMP receptors; and Class F comprises frizzled and smoothened receptors (7–10). By contrast, the GRAFS system was established based on phylogenetic analyses of human GPCRs, dividing them into five families: glutamate (G), rhodopsin (R), adhesion (A), frizzled/taste2 (F), and secretin (S). In insects, classification has primarily followed the A–F scheme (11). A significant breakthrough in insect GPCR research came with the sequencing of the *Drosophila melanogaster* genome, which provided the first comprehensive receptor inventory in an insect model (12). Since then, genomic data from more than 100 insect species, including *Anopheles gambiae*, *Aedes aegypti*, *Culex quinquefasciatus*, *Bombyx mori*, *Nilaparvata lugens*, and others, have been released (13–18). The continued expansion of genomic resources has enabled more accurate annotation and comparative analyses, offering critical insights into the evolutionary diversification of GPCRs and laying a solid foundation for elucidating their diverse roles in insect physiology and molecular biology (19, 20).

Reproduction is a fundamental biological process that underpins insect survival, population persistence, and evolutionary adaptation (21, 22). It is orchestrated by complex neuroendocrine signaling networks that regulate mating behavior, gametogenesis, fertilization, and oviposition in response to internal physiological states and external environmental conditions (23). These reproductive events are closely linked to hormonal control and energy allocation, reflecting the trade-off between reproductive investment and survival under variable ecological conditions (21, 24). Insect reproduction is orchestrated by a complex interplay of endocrine and signaling pathways, including juvenile hormone (JH), ecdysteroids, and insulin/IGF signaling (24, 25). Among these, JH signaling represents a pivotal regulatory signaling, particularly in female reproductive physiology (23, 26). Through its receptor Methoprene-tolerant (Met) and the coactivator Taiman (Tai), JH governs the transcriptional activation of genes involved in vitellogenin synthesis, follicular maturation, and ovarian development (27, 28). Although the relative contribution of these pathways varies across insect orders—for instance, JH acts synergistically with insulin signaling in Coleoptera and coordinates preparatory post-eclosion processes in Diptera—reproduction in Hemiptera is primarily under JH control (27, 29). In this order, JH functions as the principal gonadotropic signal, driving vitellogenesis and oocyte maturation, thereby establishing itself as the central endocrine regulator of female reproduction (30, 31). GPCRs play pivotal roles in insect reproduction, particularly as mediators linking JH signaling to vitellogenin synthesis and uptake (5, 32, 33). In panoistic ovary insects such as *Locusta migratoria*, systematic RNA interference (RNAi) screening has identified multiple GPCRs essential for vitellogenesis and oocyte maturation; their knockdown markedly impairs yolk protein synthesis and ovarian development (5). In the coleopteran *Tribolium castaneum*, GPCRs are critical for vitellogenin uptake into oocytes, with receptors such as *TcRh2* (Rhodopsin-like) and *TcD2R* (dopamine D2-like) being indispensable for yolk accumulation and follicle growth. Notably, *TcD2R* mediates non-genomic JH signaling, as JH treatment in heterologous systems induces dose-dependent changes in intracellular cAMP, suggesting that membrane-associated GPCRs regulate follicular patency and yolk protein uptake (33, 34). Collectively, these findings indicate that JH modulates insect reproduction through classical nuclear receptor pathways and GPCR-mediated membrane signaling (35), and this dual mechanism highlights the GPCRs as promising targets for novel reproductive interference strategies in pest management.

The brown planthopper, *N. lugens* is a highly destructive pest in rice cropping systems, characterized by strong host specificity, high fecundity, rapid development, and substantial migratory capacity (36–38). Both nymphs and adults feed on rice phloem, causing direct damage such as yellowing and wilting, as well as indirect effects including transmission of rice viruses, collectively leading to significant yield losses (39, 40). Conventional control relies heavily on chemical insecticides. However, widespread pesticide use has resulted in insecticide resistance, disruption of natural enemy populations, and unintended ecological imbalances (36, 41). Consequently, planthopper populations often undergo resurgence, manifesting as acute outbreaks in the first generation following broad-spectrum insecticide applications and as chronic outbreaks in subsequent generations induced by modern low-toxicity pesticides that stimulate reproduction, enhance nutrient availability, and alter gene expression linked to mating and oviposition (41–44). Our previous studies demonstrated that application of the fungicide jinggangmycin (JGM), commonly used to control rice sheath blight, increases glucose levels in rice plants, which in turn stimulates reproduction in *N. lugens* feeding on JGM-treated rice (41, 45). Transcriptome analysis revealed a significant upregulation of the G protein-coupled receptor GPCR A35, suggesting its potential role in regulating planthopper reproduction. In the present study, RNA interference (RNAi)-mediated silencing of *GPCR A35* confirmed that this receptor modulates reproduction by mediating JH synthesis and associated signaling pathways, which further influence ovarian development and vitellogenin accumulation in adults. These findings provide a novel molecular basis for understanding insect reproductive regulation and offer a potential target for developing environmentally friendly strategies to suppress planthopper reproduction.

## 2 Materials and methods

### 2.1 Insect culture

Populations of *N. lugens* were originally sourced from the China National Rice Research Institute (CNRRI, Hangzhou, China). Colonies were continuously maintained on rice plants (Oryza sativa cv. NanGeng 9108) under controlled environmental conditions of 27 ± 2 °C, 70 ± 10% relative humidity, and a 16:8 h light:dark cycle, following the rearing protocol described by Wu et al. (2024) (46).

### 2.2 The developmental and tissue-specific expression analysis of *GPCR A35* in *N. lugens*

Developmental samples of *N. lugens* were collected across multiple life stages, including eggs, first to fifth instar nymphs, and both female and male adults (24 and 48 h post-emergence). Each developmental stage was represented by 10–20 individuals, with 3 biological replicates. To investigate the tissue-specific expression pattern of *GPCR_A35*, six representative tissues, including the head, midgut, fat body, ovary, cuticle, and feet were dissected from 20 female adults (2 day post-eclosion) under a stereomicroscope on ice using sterilized scalpels and forceps. The selection of these tissues was based on their distinct physiological functions and potential relevance to GPCR signaling (47). Each tissue sample was collected in triplicate from 60 females. Samples were immediately frozen in liquid nitrogen and stored at -80 °C until RNA extraction. The PrimeScriptTM 1st Strand cDNA Synthesis Kit (TaKaRa, Beijing, China) produced the first-strand cDNA based on the manufacturer’s protocol. Quantitative real-time PCR (qRT-PCR) was carried out using a CFX96 real-time PCR detection system (Bio-Rad, CA, USA) in 10 μL reaction volumes containing 5 μL of 2× SYBR Premix Ex Taq II (Takara, Tokyo, Japan), 0.4 μL each of forward and reverse primers (10 μM), 1 μL of cDNA template, and 3.6 μL of nuclease-free water. The thermal cycling conditions were: 95 °C for 5 min, followed by 40 cycles of 95 °C for 10 s, 58 °C for 30 s, and 72 °C for 20 s, with a final extension at 72 °C for 10 min. A melting curve analysis was subsequently performed from 60 to 95 °C to confirm amplification specificity. Primers for *GPCR A35* were designed using Primer3Plus (https://www.primer3plus.com/) and are provided in **Supplementary Table S1**. *Nlβ-actin* served as the internal reference gene (48), and relative transcript levels were calculated using the 2^−ΔΔCT^ method (45, 49).

### 2.3 The synthesis of dsRNA and microinjection

Double-stranded RNA (dsRNA) targeting GPCR A35 was generated using primers containing T7 promoter sequences, following the strategy described by Wu et al. (2024) (46). dsRNA targeting green fluorescent protein (GFP) was synthesized as a negative control. All dsRNAs were prepared with the T7 RiboMAX™ Express RNAi System (Promega, Madison, WI, USA). PCR templates for dsRNA synthesis were amplified in a Bio-Rad thermal cycler under the following program: 35 cycles of 95 °C for 30 s, 60 °C for 30 s, and 72 °C for 45 s, with a final elongation step at 72 °C for 10 min. PCR products were purified using commercial kits (Novozymes Biotechnology, Nanjing, China) and subsequently used for dsRNA transcription. The resulting dsRNAs were stored at −80 °C until further use.

For RNAi assays, the fifth instar nymphs were anesthetized with CO_2_ and injected with 40, 60, 80, 100, and 120 ng of dsA35 or dsGFP into the mid-thorax using a Nanoject II microinjector (Drummond Scientific, PA, USA) under a stereomicroscope. Injected individuals were maintained in plastic cages (15 × 18 cm) supplied with rice seedlings at the 4-leaf stage (40). Each treatment contained at least 10 individuals and 3 independent biological replicates, and the samples were collected and flash-frozen at 48 h post-injection of dsA35 or dsGFP to determine the efficacy of RNAi.

### 2.4 Determination of JH titer and expression levels of JH signaling pathway-related genes after silencing of *GPCR A35* in female adults

Following adult emergence, female individuals derived from dsRNAs-treated nymphs were collected at 2 days post-eclosion for subsequent analyses of JH titers and the expression of JH signaling-related genes. JH levels were quantified using a commercial Juvenile Hormone ELISA Kit (Qiaodu, Shanghai, China). There were 3 biological replicates, and each replicate consisted of 10 females. Samples were weighed and homogenized in phosphate-buffered saline (PBS) at a ratio of 1 g tissue to 9 mL PBS, followed by centrifugation at 8,000 rpm for 20 min. The supernatants were collected, and the JH titer was determined according to the manufacturer’s protocol. Furthermore, the transcription level of the JH signaling-related genes (3-hydroxy-3-methylglutaryl-CoA reductase (*HMGCR*), farnesyl diphosphate synthase (*FPPS*), juvenile hormone acid methyltransferase (*JHAMT*), *Tai*, *Met*, and *Kr-h1*), *Vg*, and *VgR* were determined by qRT-PCR. The qRT-PCR was performed following the procedure described above. Reactions were performed in triplicate for each of the three biological replicates, based on three independent RNA samples, and each replicate was composed of 10 individuals. Primers for the aforementioned genes were designed using Primer3Plus (https://www.primer3plus.com/) and are listed in **Supplementary Table S1**. To assess the amplification efficiency of the aforementioned gene primers. A series of 10-fold dilutions of cDNAs from 2-day-old *N. lugens* female adults, ranging from 500 ng/µL to 0.05 ng/µL, was used to create the five-point standard curves using a linear regression model. The following equation was used to estimate the qRT-PCR amplification efficiency (E) of all genes: E = (10^[−1/slope]^ − 1) × 100% (50). The efficiencies of all tested primers and the correlation coefficient (R^2^) for each standard curve are shown in **Supplementary Table S1**.

### 2.5 The determination of protein, and the observation of female adult ovaries

After dsRNAs-injected nymphs molted into adults, the soluble proteins were extracted from reproductive tissues (fat body and ovary) of the virgin female adults at 2 days after emergence (n=15, N = 3). The Bradford method was employed to assess the soluble protein content, and BSA (Bovine Serum Albumin) was utilized to build a standard curve to determine the concentration of protein, according to Ge et al. (2020) (45). The ovaries of *N. lugens* females were isolated and observed at 2 and 4 days post-eclosion. The female adults were anesthetized with CO_2_ and subsequently dissected in 0.9% saline solution, and the fat bodies around the ovary were stripped cleanly. The isolated ovaries were washed and photographed using a microscope with a digital camera (Olympus, model SZX23, Japan). The count of ovarioles was documented using microscopic examination. There were 9 replicates used for each treatment.

### 2.6 Determine the reproductive and population parameters of *N. lugens*

The reproductive parameters of *N. lugens* were conducted following the procedures of Ge et al. (2020) (45) with minor modifications. After dsRNAs-treated fifth-instar nymphs molted into adults, newly emerged females were paired with untreated males at a 1:2 ratio in glass tubes (2.5 × 15 cm) containing tillering-stage rice stems. Control groups consisted of females injected with dsGFP or PBS. Rice stems were replaced daily during the pre-oviposition phase and every 48 h thereafter throughout the oviposition period until female death. The pre-oviposition period, oviposition duration, and number of eggs laid per female were recorded. Each treatment group included 9 replicates (dsA35♀ × control♂ vs. dsGFP♀ × control♂).

In a parallel experiment, population parameters were evaluated. Treated females were paired with untreated males and transferred onto tillering rice plants enclosed within cylindrical nylon cages (80-mesh, 20 × 80 cm). The number of F1 offspring was determined when the progeny reached the third-instar stage. Nymphs were subsequently reared in glass tubes (2.5 × 15 cm) containing rice stems until adult emergence. Meanwhile, unhatched eggs from the F0 generation were quantified to calculate the hatching rate as: offspring/(offspring + unhatched eggs). The population growth index (PGI) was calculated as F1/F0 (F0 = 4) (45).

### 2.7 Data analysis

Student’s t-tests were used to compare statistical differences in the gene expression levels and biological parameters of *N. lugens* between the control and gene-silenced groups. One-way analysis of variance (ANOVA) by the Tukey test was used to compare statistical differences in the gene expression levels of *GPCR A35* in different tissues and developmental periods. All statistical tests were conducted in SPSS 22.0 (IBM Inc., Armonk, NY, USA), and plots were generated using Origin 2023 (OriginLab Inc., Northampton, UK).

## 3 Results

### 3.1 The spatio-temporal expression profile of *GPCR A35* in *N. lugens*

**Figure 1:**
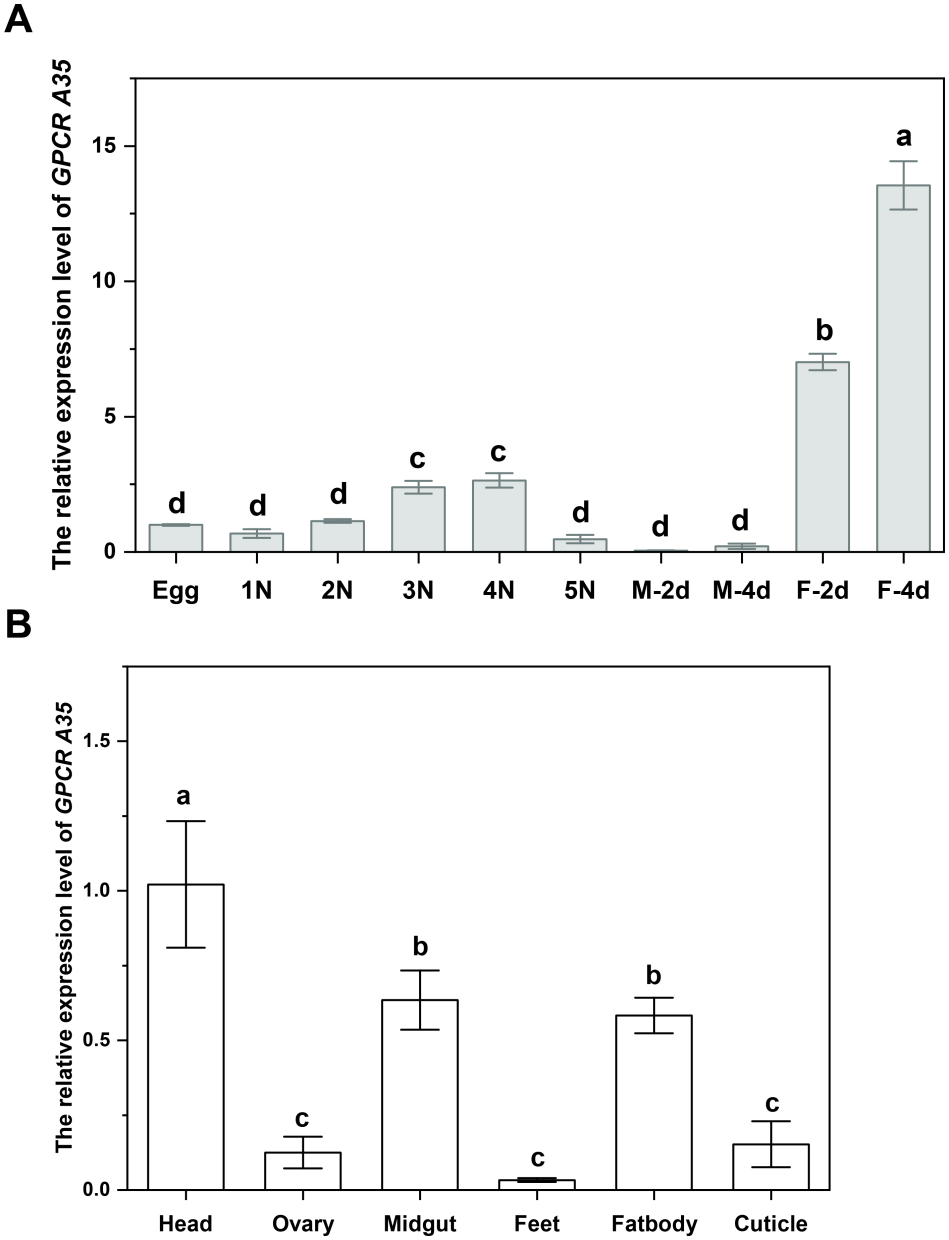
The developmental expression **(A)** and tissue-specific expression level **(B)** of *GPCR A35* in *N. lugens* females. All data are reported as means ± SE of three independent biological replications. Lowercase letters represent significant differences in *GPCR A35* expression levels among different developmental stages and tissues by the Tukey test, *P* < 0.05. .

To investigate the physiological function of GPCR A35, we first analyzed the expression pattern of *GPCR A35* in different developmental stages from egg to adult by qRT-PCR. The transcript of *GPCR A35* was expressed in the females and had the highest expression at the 4 days post-eclosion, while with only a deficient transcript level in the male adults (**Figure 1A**). Moreover, the transcript level of *GPCR A35* in six tissues (Head, Midgut, Ovary, Feet, Fatbody, and Cuticle) from the 2-day-old female adults was detected by qRT-PCR. The expression of *GPCR A35* exhibited significant tissue-specific variation in female adults. Transcript levels were highest in the head, where expression was markedly greater than in other tissues (**Figure 1B**). Moderate enrichment was observed in the midgut and fat body, whereas only low transcript abundance was detected in the ovary and feet (**Figure 1B**).

### 3.2 Effect of silencing *GPCR A35* on JH synthesis and JH signaling transduction in *N. lugens* female adults

**Figure 2:**
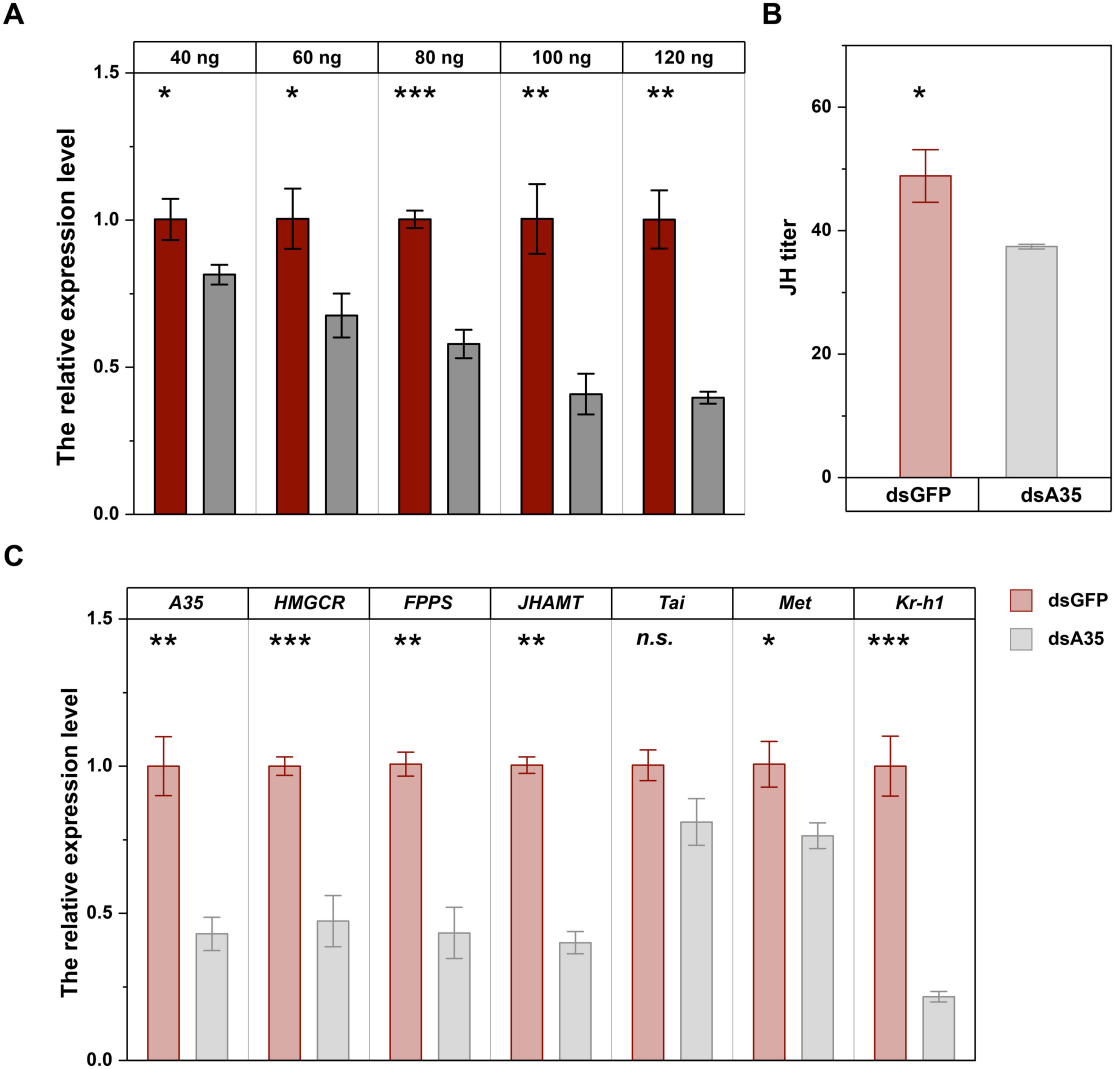
RNAi efficiency of different doses of dsA35 on *N. lugens* nymphs **(A)**, and the effects of dsA35 on JH titer **(B)** and expression levels of JH signaling-related genes **(C)**. The asterisks represent no significant differences in expression level between control (dsGFP) and treatment (dsA35) by Student’s t-test: *, *P* < 0.05; **, *P* < 0.01; ***, *P* < 0.001; *n.s.*, not significant.

Injection of dsA35 at different doses markedly suppressed *GPCR A35* expression in fifth-instar nymphs. Transcript levels were reduced by 18.7%, 32.7%, 42.3%, 59.3%, and 60.4% at 48 h after injection with 40–120 ng dsA35, respectively, compared with the dsGFP-injected nymphs (**Figure 2A**; 40 ng: *t* = 3.6, *P* < 0.05; 60 ng: *t* = 3.9, *P* < 0.05; 80 ng: *t* = 11.2, *P* < 0.001; 100 ng: *t* = 6.5, *P* < 0.01; 120 ng: *t* = 9.0, *P* < 0.01). The silencing effect increased in a dose-dependent manner, and since 100 ng dsA35 provided robust inhibition, this dose was selected for subsequent experiments. To further investigate the role of *GPCR A35* in JH signaling, the dsA35 was injected into 5th *N. lugens* nymphs. After adult emergence, qRT-PCR was employed to assess the transcript level of *GPCR A35* and JH signaling-related genes in *N. lugens* virgin females at 2 day post-eclosion (**Figure 2C**). The results showed that the silencing effect of *GPCR A35* in fifth-instar nymphs was effectively delivered to female adults, and the expression of *GPCR A35* in female adults was significantly downregulated by 57% (**Figure 2C**). Moreover, the silencing *GPCR A35* led to downregulate the JH synthetase, and JH signaling related genes expression levels, including *JHAMT*, *FPPS*, *HMGCR*, *Met*, and *Kr-h1* by 52.7% (*t* = 8.6, *P* < 0.01), 57.0% (*t* = 8.9, *P* < 0.01), 60.1% (*t* = 19.3, *P* < 0.001), 24.2% (*t* = 4.1, *P* < 0.05), and 78.3% (*t* = 11.3, *P* < 0.001) in comparison with controls at 2 day post-eclosion, respectively (**Figure 2C**), there was no significant effect on the expression levels of *Tai* (*P* > 0.05) which was downstream of the JH signal. Further, the JH titer in females at 2 days post-eclosion was significantly decreased following the knockdown of *GPCR A35* in *N. lugens* nyphmal stage (**Figure 2B**, *t* = 4.0, *P* < 0.05).

### 3.3 Silencing *GPCR A35* in the nymphal stage affects soluble protein contents and the transcript level of *Vg* and *VgR*

**Figure 3:**
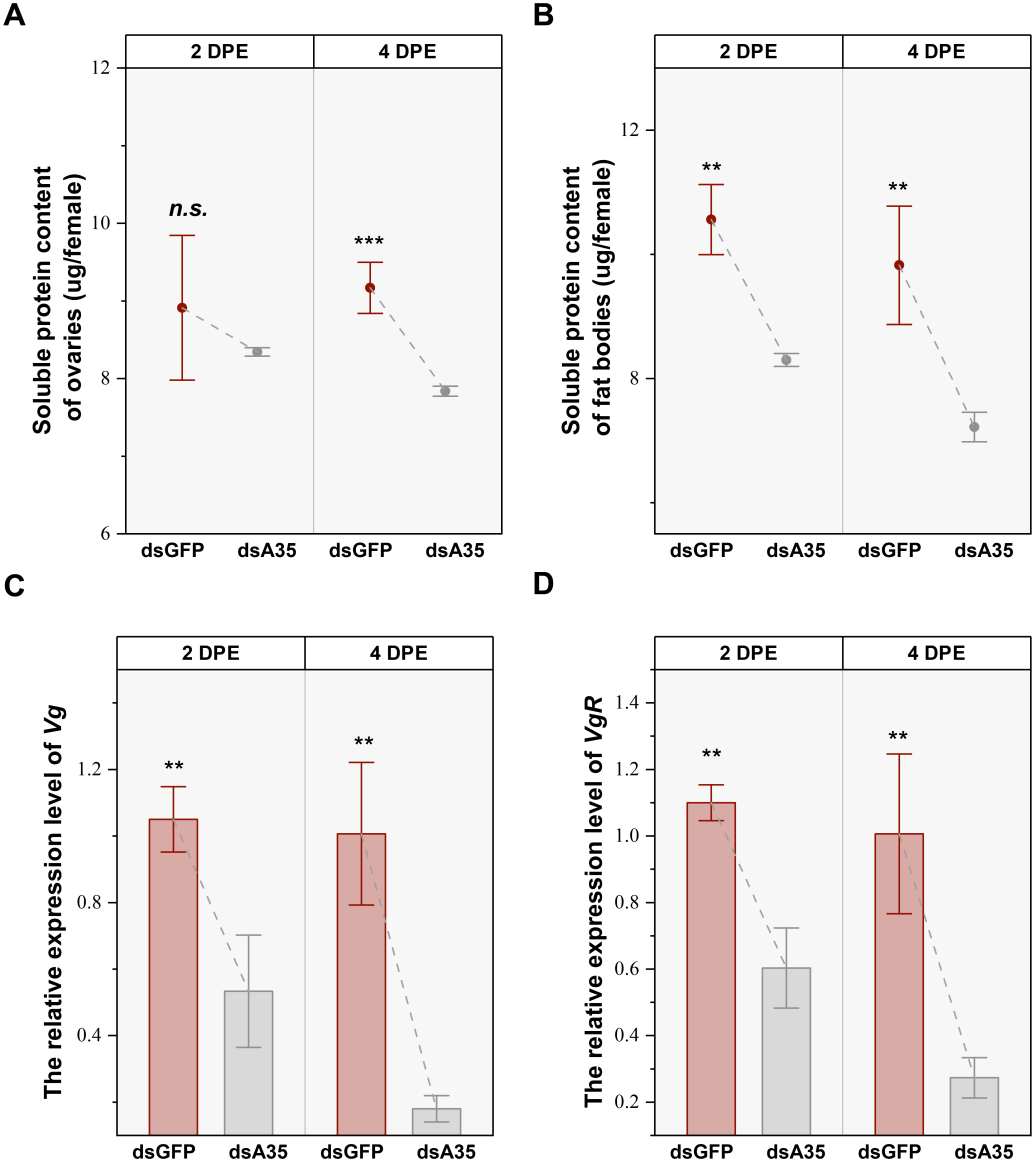
Effects of dsA35 on soluble protein content **(A, B)**, and expression patterns of Vg and VgR **(C, D)**. All data are mean ± SE. The asterisks represent statistical differences between control and treatment: **, *P* < 0.01; ***, *P* < 0.001; *n.s*., not significant. DPE, days post-eclosion.

Silencing of *GPCR A35* in the nymphal stage significantly affected protein accumulation in the reproductive tissues of virgin female adults. In the ovary, the soluble protein levels were reduced by 21.4% (*t* = 10.3, *P* < 0.01) and 26.6% (*t* = 6.9, *P* < 0.01) in females at 2 and 4 days post-eclosion, respectively, compared with dsGFP-treated females (**Figure 3A**). The protein content in fat bodies also declined, showing reductions of 14.6% (*t* = 10.3, *P* < 0.001) at 4 days post-eclosion (**Figure 3B**). In addition, knockdown of *GPCR A35* markedly suppressed the transcription of *Vg* and *VgR*. The expression levels decreased by 49.2% (*t* = 6.9, *P* < 0.01) and 45.2% (*t* = 9.8, *P* < 0.01) at 2 days post-eclosion, and decreased by 82.1% (*t* = 9.8, *P* < 0.01) and 72.9% (*t* = 7.1, *P* < 0.01) at 4 days post-eclosion, compared to dsGFP-treated females (**Figures 3C, D**).

### 3.4 Silencing *GPCR A35* inhibits ovarian development in female adults

**Figure 4:**
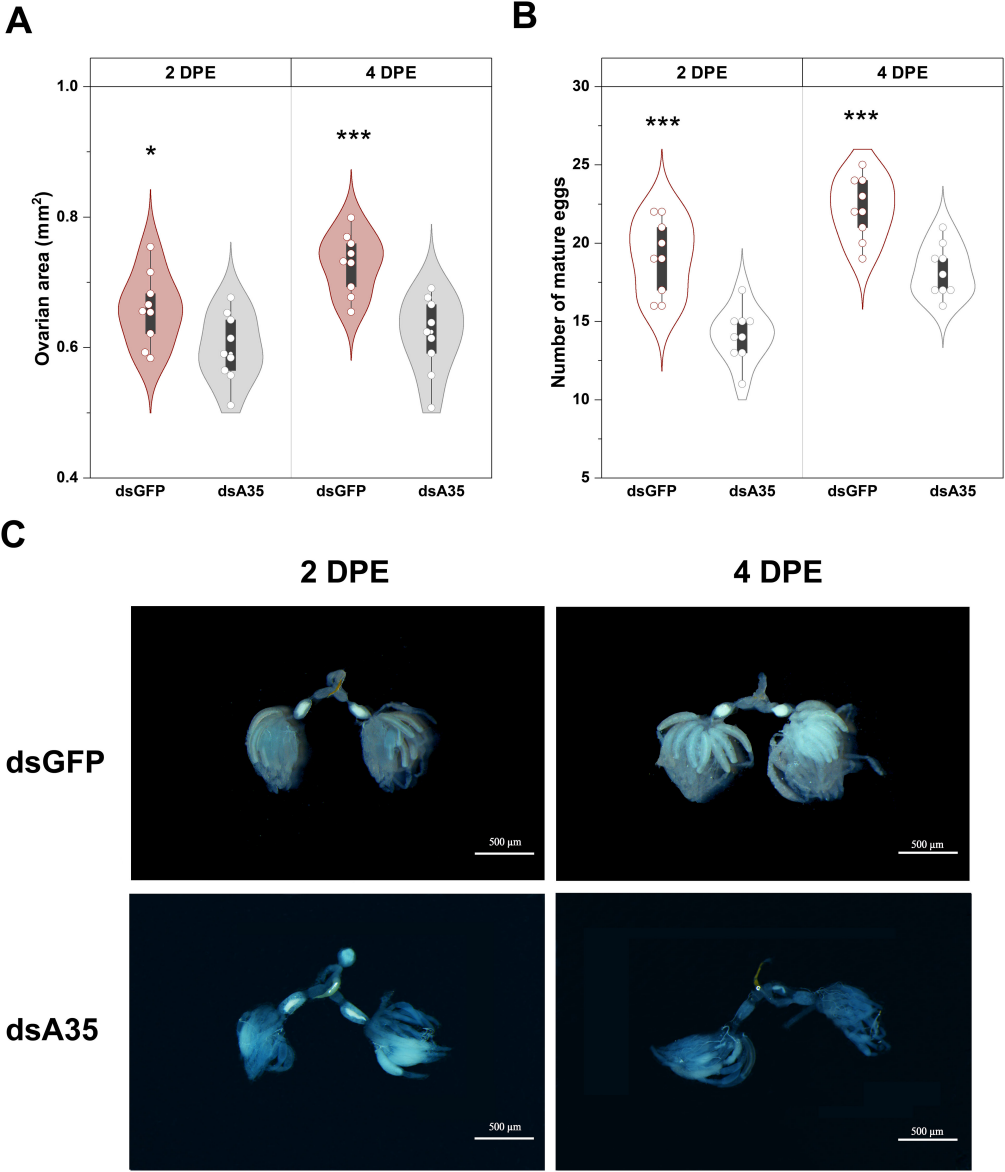
Effects of dsA35 on ovarian area **(A)**, number of mature eggs **(B)**, and ovarian development **(C)** in females.. All data are means ± SE. The asterisks represent statistically significant differences between control and treatment by Student’s t-test: *, P < 0.05; ***, P < 0.001. DPE, days post-eclosion.

To further elucidate the role of GPCR A35 in the female reproductive process of *N. lugens*, dsRNAs targeting *GPCR A35* or *GFP* were injected into fifth-instar nymphs, and ovarian morphology was subsequently examined in virgin females at 2 and 4 days post-eclosion (**Figure 4C**). In control females (dsGFP-injected), ovaries developed normally, displaying abundant, well-formed ovarioles filled with mature eggs at 4 days post-eclosion. In contrast, knockdown of *GPCR A35* resulted in markedly impaired ovarian development (**Figure 4C**). Ovaries from dsA35-treated females exhibited delayed growth, with only a limited number of fully elongated, banana-shaped ovarioles and fewer mature oocytes at 4 days post-eclosion. Quantitative analysis further confirmed these morphological observations: ovarian area and mature oocyte numbers were significantly reduced in dsA35-injected females, with decreases of 9.0% (*t* = 2.3, *P* < 0.05) and 26.2% (*t* = 5.2, *P* < 0.001) at 2 days post-eclosion, and 15.1% (*t* = 4.4, *P* < 0.001) and 18.0% (*t* = 4.7, *P* < 0.001) at 4 days post-eclosion, respectively, compared with dsGFP-treated females (**Figures 4A, B**).

### 3.5 Silencing of *GPCR A35* impairs reproductive performance and population growth in *N. lugens*

Finally, we assessed the impact of *GPCR A35* knockdown on reproductive performance and population parameters of *N. lugens* (**Table 1**). The results showed that silencing of *GPCR A35* markedly extended the pre-oviposition period (*t* = 3.7, *P* < 0.05) and shortened the oviposition duration (*t* = 2.7, *P* < 0.05) in female adults, accompanied by a dramatic 51.3% reduction in fecundity compared with dsGFP-injected controls. Moreover, suppression of *GPCR A35* during the nymphal stage (F0 generation) significantly impaired population-level traits in the F1 generation. Specifically, the population growth index (PGI) was reduced by 46.8% (*t* = 5.4, *P* < 0.05), respectively, relative to controls (**Table 1**). These findings demonstrate that suppression of *GPCR A35* not only compromises female reproductive performance but also exerts a reduction on population expansion.

**Table 1:** Effects of silencing *GPCR A35* on the reproductive and population parameters of *N. lugens*.

Reproductive and population parameters	dsGFP	dsA35
Number of eggs laid	311.22 ± 19.72 a	246.8 ± 47.4 b
Pre-ovipositon periods	4.33 ± 0.17 a	3.44 ± 0.18 b
Ovipositon periods	16.22 ± 0.94 a	13.22 ± 0.60 b
Female longevity	19.11 ± 0.87 a	15.00 ± 0.87 b
Hatching rate	0.71 ± 0.02 a	0.66 ± 0.03 a
Sex ratio	1.46 ± 0.14 a	1.47 ± 0.18 a
Population growth index	198.22 ± 16.19 a	105.44 ± 5.41 b

All data are means ± SE. The different lowercase letters represent statistically significant differences (*P* < 0.05) between control and treatment by Student’s t-test.

## 4 Discussion

GPCRs defined by their conserved seven-transmembrane (7TM) architecture, represent the largest and most versatile family of cell surface receptors (51). Despite their structural homogeneity, GPCRs exhibit remarkable ligand diversity, being able to detect a wide spectrum of extracellular signals, including photons, odorants, neurotransmitters, and hormones (52). This functional plasticity allows GPCRs to orchestrate a broad range of physiological and biochemical processes, such as sensory perception (vision, olfaction, and taste), regulation of behavior and mood, modulation of immune responses, and control of cell growth and proliferation (53–55). Studies have revealed specific GPCR genes and their potential biological functions that may impact insects physiology (4, 20, 56, 57), including their reproduction (33, 58, 59), and regulating their growth and development (57, 60–62), as well as their stress responses (63–66), their feeding patterns (67, 68), their other behaviors (69–71), and many other physiological processes (47, 56). Moreover, increasing evidence suggests that these receptors play pivotal roles in reproductive regulation and are even implicated in gametogenesis, fertilization, and oviposition, underscoring their broad and multifaceted biological significance. In oviparous insects, reproduction is a complex process involving multiple sequential stages, including previtellogenesis, vitellogenesis, chorion formation, oocyte maturation, and egg hatching (72). Sexual reproduction is essential for insect survival and evolutionary success, as it generates genetic diversity and ensures population continuity. Recent study has highlighted that insect GPCRs also play crucial roles in modulating endocrine signaling (47). The neuropeptide GPCRs, such as allatotropin and allatostatin receptors, are expressed in the corpora allata and regulate JH production through G protein–mediated second messenger pathways, including cAMP and Ca²^+^ signaling cascades, suggesting that membrane-bound GPCRs can influence hormonal homeostasis either by activating intracellular signaling that affects JH synthesis or by indirectly modulating the downstream JH receptor complex (3, 35, 47, 73, 74).

*N. lugens* is a major rice pest characterized by its high fecundity and overlapping generations, enabling rapid population development within a single cropping season (75, 76). A previous study in *N. lugens* identified 48 neuropeptide precursors and about 57 candidate GPCRs (77). Most of these receptors belong to typical rhodopsin- and secretin-like GPCR families, but several unusual receptor types have also been uncovered, suggesting a complex neuroendocrine system in this species. Expression profiling revealed pronounced stage- and tissue-specific patterns, implicating these receptors in diverse physiological functions including development, reproduction, feeding, and stress responses (77, 78). Functional studies showed that elevenin receptor *NlA42* regulates cuticular pigmentation, and several octopamine receptors are associated with reproductive regulation and stress adaptation (79). Nevertheless, the majority of *N. lugens* GPCRs remain functionally uncharacterized, and their downstream signaling pathways are still poorly understood. Our previous studies demonstrated that spraying the fungicide JGM increases glucose content in rice plants, which in turn stimulates reproduction in *N. lugens* (41, 45). Transcriptome analysis revealed a significant upregulation of the GPCR A35, suggesting its potential role in reproduction. In this study, GPCR A35 was identified in *N. lugens*, and it is highly expressed in females at 4 days post-eclosion, with enrichment in the head and fat body, while it is less expressed in the ovary and feet. The predominant expression of *GPCR A35* in the head, rather than in the ovaries, suggests that it may regulate reproduction indirectly through neuroendocrine pathways rather than by acting directly within ovarian tissues. Considering that JH is synthesized in the corpora allata (23, 31) located near the brain, the head-enriched expression of *GPCR A35* is consistent with its potential role in modulating JH biosynthesis and signaling. Similar distribution patterns have been reported in other insects such as *Drosophila*, *Bombyx mori*, *Solenopsis invicta*, and *T. castaneum*, indicating that GPCRs are predominantly distributed in the insect brain and central nervous system because they serve as primary receptors for neuropeptides and biogenic amines, coordinating neuroendocrine and behavioral functions. (17, 34, 58, 59, 80–82). Their enriched expression underscores their essential roles in regulating behavior, reproduction, feeding, stress responses, and other vital physiological functions, highlighting the centrality of GPCR-mediated signaling to insect development and adaptation (47). The insect fat body is a key metabolic and endocrine organ that synthesizes and stores nutrients, vitellogenin, and hormones, thereby providing the essential energy reserves and signaling molecules required for oocyte maturation and successful reproduction (83). Enrichment of *GPCR A35* in the fat body suggests its potential involvement in the reproductive processes of the *N. lugens*.

In most insects, vitellogenesis, which is a central event of female reproduction, involves the production and secretion of vitellogenin (Vg) and other yolk protein precursors (YPPs) by the fatbody, followed by internalization of YPPs by maturing oocytes through receptor-mediated endocytosis (24, 84). Reproduction in hemimetabolous and Hemiptera is governed by JH (29, 85, 86). JH is a key insect steroid hormone that plays vital roles in female reproduction, particularly in regulating oogenesis and embryogenesis. During oogenesis, JH promotes vitellogenin synthesis in the fat body and facilitates its uptake into oocytes via vitellogenin receptors, thereby supporting yolk accumulation and oocyte maturation (30, 32, 87). In this study, RNAi-mediated silencing of *GPCR A35* in fifth-instar nymphs was effectively delivered to female adults, leading to altered expression of JH synthase and a consequent reduction in JH titer. This disruption further affected the downstream JH signaling pathway by downregulating *Met* and *Kr-h1* expression, ultimately decreasing protein content in the female reproductive tissues and suppressing the expression of *Vg* and *VgR*. As a result, reproductive performance and the number of offspring were significantly reduced in *N. lugens*. JH is a key endocrine regulator that controls insect reproduction by modulating vitellogenesis, oocyte maturation, and reproductive organ development. Increasing evidence suggests that GPCRs participate in the modulation of JH biosynthesis and signal transduction, thereby influencing reproductive processes through neuroendocrine regulation and cross-talk with the JH pathway. A neuropeptide GPCR was significantly overexpressed in the corpora cardiaca and brain of *B. mori*, indicating the potential involvement of JH biosynthesis processes (88). The overexpression of an allatotropin GPCR receptor (*AeATr*) gene was characterized in the nervous system and corpora alata-corpara cardiac complex of *Aedes aegypti*. Blood feeding depressed the transcript level of *AeATr*, and was associated with JH biosynthesis in mosquitoes (74). In the bumblebee, *Bombus terrestris*, an allatotropin GPCR has been identified that is overexpressed in the male bumblebee accessory glands, predicting its potential involvement in JH biosynthesis (73). A-type allatostatin neuropeptides and GPCRs have been discovered in JH biosynthesis in many insect species, including *Drosophila*, cockroaches, crickets, and termites (8). In *L. migratoria*, among the 22 GPCRs identified in the ovarian transcriptome, *LGR4*, *OR-A1*, *OR-A2*, *Mthl1*, *Mthl5*, and *Smo* showed the highest expression in the ovary. RNAi screening revealed that silencing six GPCRs caused defective phenotypes characterized by disrupted vitellogenin accumulation in developing oocytes, arrested ovarian development, and impaired oocyte maturation. Specifically, *LGR4* and *Oct/TyrR* appeared to regulate Vg synthesis in the fat body, whereas *OR-A1*, *OR-A2*, *mAChR-C*, and *CirlL* were involved in Vg transport and uptake. These results provide important insights into the regulatory roles of GPCRs in JH-mediated reproductive processes in insects (5).

The JH plays a pivotal role in regulating insect metamorphosis and reproduction (89). JH exerts its reproductive control through its receptor Met, which forms a heterodimer with the bHLH-PAS protein Tai. Within the nucleus, the Met–Tai complex binds to specific promoter regions known as JH response elements (JHREs) to regulate target gene transcription (21, 24). Although Tai does not directly bind JH, the JH-Met interaction facilitates their dimerization and subsequent nuclear translocation of Met, a key step in JH signaling (28, 90). Following the identification of Met as the JH receptor, the JH–Met–Kr-h1 regulatory model was established, in which *Kr-h1* acts downstream of Met to mediate JH-dependent reproductive functions (91). In this study, suppression of *GPCR A35* further downregulated JH signaling pathway by inhibiting *Met* and *Kr-h1* expression. In *Helicoverpa armigera*, RNAi-mediated silencing of GPCRs downregulated the expression of *Kr-h1*, which further affected larval growth and development, and GPCRs are also involved in JH III-induced broad isoform 7 (BrZ7) phosphorylation (92, 93). In *L. migratoria*, JH activated multiple intracellular signaling pathways involving *GPCR*, *RTK*, *PLC*, and *IP3R*, which phosphorylate the Na^+^/K^+^-ATPase subunit at amino acid residue Ser^8^, consequently activating Na^+^/K^+^-ATPase for the induction of patency in vitellogenic follicular epithelium (94). Additionally, JH triggers a cascade comprising *GPCR*, *PLC*, extracellular Ca²^+^, and *PKC*, leading to *VgR* phosphorylation and facilitating Vg endocytosis (25). Another JH-induced pathway involves GPCR, which showed that JH acts via the GPCR-Cdc42-aPKC signaling cascade that triggers the phosphorylation of Par3, a critical scaffold protein of zonula adherens. JH-dependent Par3 phosphorylation results in its dissociation from the β-Catenin/E-Cadherin complex, consequently leading to the opening of patency for Vg transport (26). In *T. castaneum*, dopamine GPCR-mediated JH signaling promotes Vg accumulation and elevates cAMP levels in oocytes (33). Moreover, numerous studies have demonstrated that the silencing of *Met*, *Tai*, and *Kr-h1* caused downregulation of *Vg*, inhibition of ovarian maturation, and a decrease in the offspring population and egg hatching rate (31, 95–98). In *N. lugens*, *Met* and *Kr-h1* were critical in ovarian development and oocyte maturation (95, 99). In the current study, suppression of *GPCR A35* downregulated the expression of *Kr-h1, which* might affect *Vg* transcription and arrest ovarian development in *N. lugens* females. Finally, we further found that silencing *GPCR A35* led to shortened oviposition durations, decreased fecundity, and a decline in offspring.

In conclusion, this study functionally characterized a GPCR A35, in *N. lugens*. *GPCR A35* was predominantly expressed in female heads and fat bodies and proved to be essential for reproduction. RNAi-mediated silencing of *GPCR A35* reduced JH biosynthesis and signaling activity, leading to the suppression of *Vg* and *VgR* expression, impaired ovarian development, and a marked decline in fecundity. Collectively, these results indicate that GPCR A35 regulates female reproductive capacity by modulating JH-mediated vitellogenesis and oogenesis. This work provides new insights into GPCR-mediated endocrine regulation in hemipteran insects and identifies GPCR A35 as a potential molecular target for RNAi-based green pest management strategies.

## Data Availability

The raw data supporting the conclusions of this article will be made available by the authors, without undue reservation.
