# Supplementary material for: The GPCR A35 regulates fecundity of Nilaparvata lugens Stål via juvenile hormone signaling
Source: Front Insect Sci. 2025 Nov 26;5:1719937. doi: 10.3389/finsc.2025.1719937 (PMC12689387; doi:10.3389/finsc.2025.1719937)
Supplement: Supplementary file 1 [file Table1.docx]

Supplementary Material:

| Purpose | Primer name | Primer sequence（5’-3’） | Efficiency (%) | R^2^ |
| --- | --- | --- | --- | --- |
| qRT-PCR | Q- *A35*-F | TTCCGAGATGCTTTCAAGGGTA | 106.90 | 0.98 |
|  | Q- *A35*-R | CGAGTGGTTCAGCAGAGTGGG |  |  |
|  | Q- *HMGCR-*F | CATTGGCAGCCGGCCATCTA | 117.35 | 0.98 |
|  | Q- *HMGCR-*R | CCGCAACTCAAAGCCTGGGA |  |  |
|  | Q- *FPPS-*F | TGACTCTGGAGCCCATCCCG | 110.14 | 0.99 |
|  | Q- *FPPS-*R | AACGAGCAACGGGAAAGCGA |  |  |
|  | Q- *JHAMT-*F | TGCAGTGAACCCGTTCTTGGG | 106.99 | 0.98 |
|  | Q- *JHAMT-*R | ACAACCACTCGGTTGGGCTG |  |  |
|  | Q- *Met-*F | AGCTATTCCTGAACGCCGCC | 118.15 | 0.98 |
|  | Q- *Met-*R | CTCTTGGTTGGTGGGCGTGT |  |  |
|  | Q- *Tai-*F | ATGATCCCAACCACTTCAGC | 126.79 | 0.99 |
|  | Q- *Tai-*R | TTCCACTCACACTACCACCA |  |  |
|  | Q- *Kr-h1-*F | AGTGCCTGCAGCAAAGCAGA | 116.61 | 0.98 |
|  | Q- *Kr-h1-*R | CGAGGATGACGGCACAGGAC |  |  |
|  | Q-*Vg*-F | CCCTAGTCAGTCCAGTGCC | 116.67 | 0.98 |
|  | Q-*Vg-*R | TGGCGAGAGGAACTGTCA |  |  |
|  | Q-*VgR*-F | AGGCAGCCACACAGATAACCGC | 121.16 | 0.99 |
|  | Q-*VgR-*R | AGCCGCTCGCTCCAGAACATT |  |  |
|  | *β-actin*-F | CCGTCCCCATCTATGAAGGTT | 109.06 | 0.99 |
|  | *β-actin*-R | GCGGTCGTCGTGAAGGAGTAAC |  |  |
| dsRNA synthesis | T7-*A35*-F | TAATACGACTCACTATAGGG  AGAGATGACATTATTGCCCGTTCC |  |  |
|  | T7-*A35*-R | TAATACGACTCACTATAGGG  AGACCGTGATGGTCAACACCGTAG |  |  |
|  | T7-*GFP*-F | TAATACGACTCACTATAGGG  AAGGGCGAGGAGCTGTTCACCG |  |  |
|  | T7-*GFP*-R | TAATACGACTCACTATAGGG  CAGCAGGACCATGTGATCGCGC |  |  |
